# Bovine Lactoferrin Inhibits Toscana Virus Infection by Binding to Heparan Sulphate

**DOI:** 10.3390/v7020480

**Published:** 2015-01-29

**Authors:** Agostina Pietrantoni, Claudia Fortuna, Maria Elena Remoli, Maria Grazia Ciufolini, Fabiana Superti

**Affiliations:** 1 Department of Technology and Health, Istituto Superiore di Sanità, Viale Regina Elena, 299, Rome 00161, Italy; E-Mail: agostina.pietrantoni@iss.it; 2 Department of Infectious, Parasitic and Immune-mediated Diseases, Istituto Superiore di Sanità, Viale Regina Elena, 299, Rome 00161, Italy; E-Mails: claudia.fortuna@iss.it (C.F.); mariaelena.remoli@iss.it (M.E.R.); mariagrazia.ciufolini@iss.it (M.G.C.)

**Keywords:** Toscana virus, lactoferrin, antiviral agents, Phlebovirus

## Abstract

Toscana virus is an emerging sandfly-borne bunyavirus in Mediterranean Europe responsible for neurological diseases in humans. It accounts for about 80% of paediatric meningitis cases during the summer. Despite the important impact of Toscana virus infection-associated disease on human health, currently approved vaccines or effective antiviral treatments are not available. In this research, we have analyzed the effect of bovine lactoferrin, a bi-globular iron-binding glycoprotein with potent antimicrobial and immunomodulatory activities, on Toscana virus infection *in vitro*. Our results showed that lactoferrin was capable of inhibiting Toscana virus replication in a dose-dependent manner. Results obtained when lactoferrin was added to the cells during different phases of viral infection showed that lactoferrin was able to prevent viral replication when added during the viral adsorption step or during the entire cycle of virus infection, demonstrating that its action takes place in an early phase of viral infection. In particular, our results demonstrated that the anti-Toscana virus action of lactoferrin took place on virus attachment to the cell membrane, mainly through a competition for common glycosaminoglycan receptors. These findings provide further insights on the antiviral activity of bovine lactoferrin.

## 1. Introduction

Toscana virus (TosV) (family *Bunyaviridae*, genus Phlebovirus) is an enveloped, negative-stranded RNA virus, with a genome consisting of three segments: small (S), medium (M), and large (L), encoding the nucleoprotein (N) and a non-structural protein (NSs), the envelope glycoproteins (GN and GC), and a non-structural protein (NSm), and the large protein (L, the viral RNA-dependent RNA polymerase), respectively [1,2]. It was first isolated in 1971 from sandfly, *Phlebotomus perniciosus*, collected in the Toscana region of Central Italy [3,4]. Following its discovery, several other regions of Italy, where the insect vectors (*Phlebotomus perniciosus* and *Phlebotomus perfiliewi*) are present [5], showed to be endemic. Although TosV rarely gives rise to clinical infection, in these regions it was responsible for neuroinvasive infections during the summertime [6,7,8,9,10]. Moreover, other Mediterranean Countries, including Spain, France, Portugal, Cyprus, Greece, and Turkey, have been included in the endemic regions of TosV [11]. TosV represents the only sandfly-transmitted virus that demonstrates neurotropic activity to date [12,13], particularly during the summer, with a peak in August, correlating with the activity of phlebotomus vectors [14,15,16,17,18,19,20]. Infections without central nervous system involvement have also been described [15].

TosV clinical infection starts as a mild febrile illness, following an incubation period of three to seven days, without involvement of the central nervous system (CNS) [5]. Neuroinvasive infections usually begin with headache, fever, nausea, vomiting, and myalgia. Physical examination may show neck rigidity, Kernig sign, and, in some cases, unconsciousness, tremors, paresis, and nystagmus. The outcome is usually favorable without significant sequelae. Other clinical manifestations have been reported, such as encephalitis [21], severe meningo-encephalitis [22], testicular involvement [23], deafness [24,25], persistent personality alterations [26], long-lasting unconsciousness with seizures, prolonged convalescence [27], and even fatal encephalitis [28].

Despite the important impact of TosV infection-associated disease on human health, there are currently no available approved vaccines or specific antiviral therapies for this disease.

Bovine lactoferrin (bLf) is a glycoprotein, consisting of a single polypeptide chain of 689 amino acidic residues, with a molecular mass of about 80 kDa, which binds two iron atoms with very high affinity [29]. Such a protein has an alkaline isoelectric point (about pI 9) and its cationic nature could have a major role in the ability to bind cells and many anionic molecules, such as glycosaminoglycans (GAGs) and lipopolysaccharide. BLf is present in various biological fluids and in specific granules of polymorphonuclear leukocytes [30], and possesses a variety of biological functions, such as promotion of iron absorption, immunomodulation, and inhibiting activity towards different pathogens [31,32,33,34]. In particular, bLf has been recognized as potent inhibitor of different enveloped viruses, such as human cytomegalovirus [35,36], Herpes Simplex Viruses types 1 and 2 [37,38,39,40], Human immunodeficiency virus [35], Human hepatitis C virus [41], Hantavirus [42], Hepatitis B virus [43], Respiratory syncytial virus [44], Influenza virus [45,46], Flavivirus [47], and Alphavirus [48].

In an effort to identify antiviral therapies effective against TosV, in this research we have analyzed the effect of bLf on virus infection *in vitro.* Our results indicate that bLf treatment specifically inhibits viral cytopathic effect and that its action takes place in an early phase of infection. In particular, bLf has been found to prevent viral infection by binding to GAGs, which, in turn, can act as cell receptors for TosV.

## 2. Results

### 2.1. Activity of bLf on TosV Cytopathic Effect

**Figure 1:**
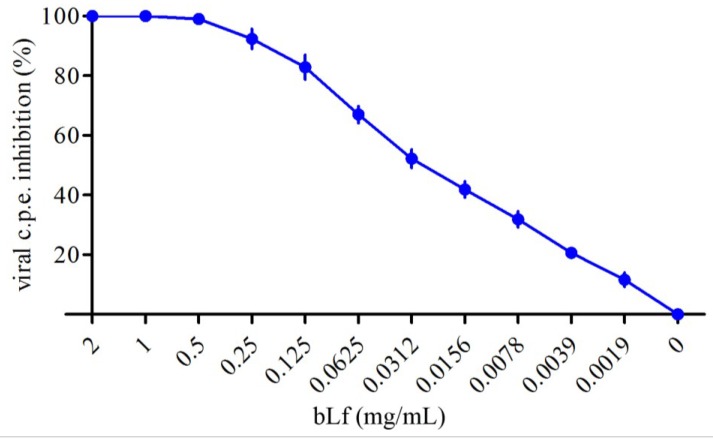
Dose-response curve of bLf toward TosV cytopathic effect in Vero cells. Vero cells were infected with virus (m.o.i. 1). BLf was incubated at different concentrations with the cells before (1 h at 37 °C) and during (1 h at 37 °C) the viral absorption step and newly added after the removal of virus inoculum. At 48 h post-infection, the percentage of c.p.e. was evaluated. Data showed represent the mean of at least quadruplicate samples.

A preliminary set of experiments was carried out to determine the maximal non-cytotoxic concentration of bLf. For this purpose, twofold serial dilutions of protein from 2 mg/mL in DMEM were incubated with Vero cells for 48 and 72 h at 37 °C. Under these conditions, bLf did not affect any of the cytotoxicity parameters up to the highest dose. To establish whether bLf could inhibit TosV cytopathic effect (c.p.e.) in Vero cells, two-fold serial dilutions of lactoferrin, starting from the highest non-cytotoxic concentration, were incubated with the cells through the infection (before, during, and after virus adsorption). In our experimental conditions, bLf showed dose-dependent inhibitory activity (Figure 1).

### 2.2. Effect of bLf on Different Steps of Viral Infection

**Figure 2:**
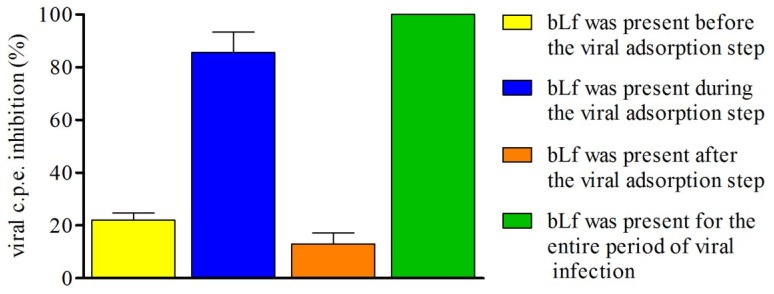
Effect of bLf on different steps of TosV infectious cycle. Monolayers of Vero cells were infected with TosV (m.o.i. 1) and treated with bLf (1mg/mL) during different phases of infection: (**A**) before virus inoculation; (**B**) during virus inoculation; (**C**) after virus inoculation; and (**D**) throughout infection. After 48 h of infection the viral c.p.e. was measured by neutral red uptake assay. Data showed represent the mean of at least quadruplicate samples.

To ascertain whether the antiviral effect of bLf took place on viral adsorption or at a different step of viral replication, the inhibiting activity of protein (1 mg/mL) was assessed by following different experimental procedures: (i) the cells were incubated with bLf (1 h at 37 °C), washed three times with medium and then infected (1 h at 37 °C); (ii) bLf was added, together with the virus inoculum, during the adsorption step (1 h at 37 °C); (iii) bLf was incubated with the cells after the viral adsorption step (48 h at 37 °C); (iv) bLf was present during the whole experiment. TosV c.p.e. was measured by neutral red uptake assay. Results reported in Figure 2 show that a high inhibition was obtained under the conditions used in procedures (ii) and (iv).

### 2.3. Neutralization of TosV Infection by Heparin

Our results indicated that the anti-TosV activity of bLf is mainly directed on viral attachment to target cells. It has been reported that GAGs, like heparin and heparan sulphate (HS), participate to the attachment of bLf to target cells [49]. Moreover, Rift Valley fever virus (RVFV), an emerging arthropod-borne pathogen belonging to the Phlebovirus genus of the *Bunyaviridae* family, has been demonstrated to bind to GAGs for entry susceptible cells [50]. Our hypothesis was that, if TosV also requires GAGs for the attachment to and entry into the target cells, bLf inhibitory activity might be due to a competition with the virus for a common receptor.

**Figure 3:**
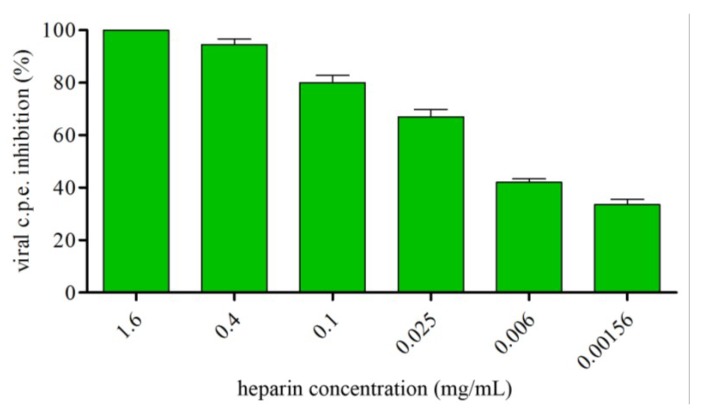
TosV infection is decreased in the presence of heparin. TosV was incubated with different concentrations of heparin for 1 h at 37 °C in culture medium prior to infection of Vero cells. At 48 h post infection c.p.e. was measured by neutral red uptake assay. The data shown correspond to the results of a representative set of two independent experiments performed in quadruplicate.

To explore whether GAGs are used for viral attachment, we examined whether, and to what extent, heparin, often used experimentally as an HS analog, could affect virus replication. This was tested by neutral red staining measuring of virus-infected Vero cells as a measure of cell viability. Results obtained showed that viral cytopathic effect was markedly inhibited by pre-incubation with heparin with a dose-dependent relationship (Figure 3).

### 2.4. Modulation of Infectivity by Enzyme Treatment of Cell Surface GAGs

To confirm the role of GAGs on TosV-cell interactions, further experiments were carried out based on the hypothesis that if cell-surface GAGs modulate infection, their enzymatic digestion should decrease the viral infectivity. Cells were incubated with heparinase I, specific for heparin and highly sulfated domains, heparinase II, specific for heparin and heparan sulfate, and heparinase III, specific for heparan sulfate [51].

**Figure 4:**
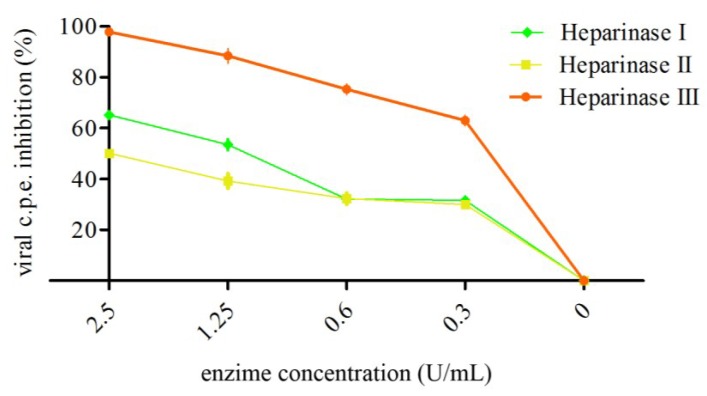
Modulation of infectivity by enzyme treatment of cell surface GAGs. GAGs were enzymatically removed from the cell surface of Vero cells by treating the cells for 1 h at 37 °C with heparinase I, II, or III at the indicated concentrations. The cells were washed twice with culture medium and then infected with TosV for 1 h at 37 °C. After 48 h of infection the viral c.p.e. was measured by neutral red uptake assay. The data shown correspond to the results of a representative set of two independent experiments performed in quadruplicate.

Treatment of Vero cells with each of the heparinases for 1 h at 37 °C was found to reduce viral infection, significantly, in a dose-dependent manner. In particular, treatment of cells with heparinases I, II, and III at 2.5 U/mL significantly inhibited TosV infection by 65.2% ± 6.6%, 50.1% ± 4.8%, and 97.6% ± 2.0%, respectively (Figure 4).

These results confirm that HS moiety of GAGs expressed on the cell surface intervenes in TosV-mediated infection.

**Figure 5:**
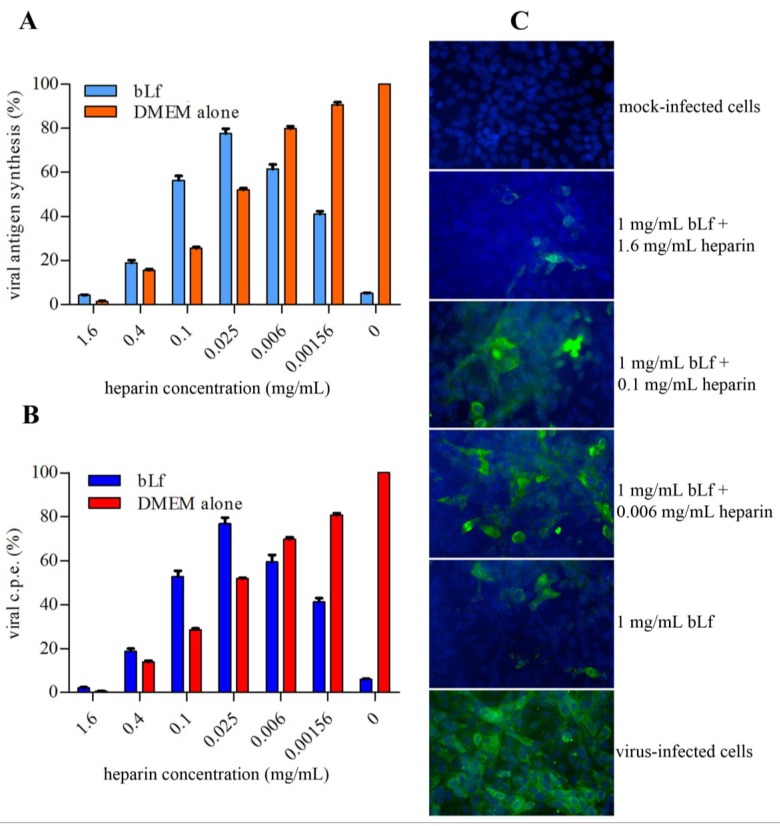
Effect of heparin and bovine lactoferrin (bLf) in combination on TosV infection. BLf (1mg/mL) and heparin, one at a time or mixed together, were incubated with Vero cells during the virus adsorption step (1 h at 37 °C). (**A**) After 18 h incubation at 37 °C, viral replication was monitored by immunofluorescence and expressed as percentage of viral antigen synthesis; (**B**) After 48 h incubation at 37 °C, the percentage of viral c.p.e. was measured by neutral red uptake assay. Data showed represent the mean of at least quadruplicate samples. (**C**) Representative pictures of fluorescence microscopy. Nuclei were counterstained with 4’,6’diamidino-2-phenylindole (DAPI) (magnification, **×**40).

### 2.5. Effect of Lactoferrin and Heparin on TosV Infection

As bLf binds GAGs directly, we investigated whether heparin could compete with the activity of bLf against TosV infection. To test the anti-TosV effect of both heparin and lactoferrin in our virus-cell system, bLf (1 mg/mL) and different concentrations of heparin, one at a time or mixed together, were incubated with Vero cells during the TosV adsorption step (1 h at 37 °C).

After 18 h incubation at 37 °C, viral antigen synthesis was monitored by immunofluorescence and expressed as percentage of infected cells (Figure 5A). After 48 h incubation at 37 °C, the c.p.e. was measured by neutral red uptake assay (Figure 5B).

The results obtained showed that, like lactoferrin, heparin reduced both viral antigen synthesis and c.p.e. in a dose-dependent manner and that at the highest heparin concentration the inhibitory activity on TosV infection was due to heparin and was not modified by the presence of lactoferrin. When the heparin concentration was reduced to 25 µg/mL, the inhibitory effect of both compounds was partially abolished. When the heparin concentrations became low (6 µg/mL), the inhibitory effect of bLf was increased, again being evident in the presence of 1.56 µg/mL of heparin.

## 3. Discussion

Lactoferrin exhibits inhibitory activities against a wide range of viruses [52] and it has been reported that human and bovine lactoferrins inhibit the infection of mosquito-borne viruses, such as Sindbis virus and Semliki Forest virus [53], Japanese encephalitis virus [47], and Mayaro virus [48].

TosV, the sandfly-borne phlebovirus with the greatest known virulence for humans, possesses a marked tropism for central and peripheral neurological systems and is cause of aseptic meningitis, meningoencephalitis, and encephalitis during the warm seasons in countries in which it circulates [20,21,22]. The probable underestimation of its spread, due to the high percentage of febrile or asymptomatic cases, makes this virus a “neglected human pathogen”. It is known today that TosV circulation is larger than initially believed. Despite increasing evidence of the role of TosV in human public health, up to now there are no available, approved vaccines or specific antiviral therapies for this disease. For this reason, the discovery and/or development of new drugs active against this virus may be useful to reduce the effects of the infection in the most severe forms.

In this paper, we analyzed the activity of bLf against TosV infection in Vero cells. Our results showed that bLf strongly inhibited infection of cells by TosV in a dose-dependent manner. As a matter of fact, at a concentration of 31.25 μg/mL bLf caused a 50% reduction in viral c.p.e. Regarding the bLf effect on the different steps of viral infection, we observed that the protein mainly acts at the level of cell attachment, interfering with virus-cell binding rather than on virus replication after the cells becomes infected. These results are in agreement with the majority of the studies on the antiviral activity of bLf, suggesting that the protein prevents virus entry into cells instead of later phases of viral replication.

BLf can act on virus cell entry by binding to the viral particles or by binding to host cell molecules that viruses use either as receptors or co-receptors [33]. In this context, binding of bLf to cell-surface heparan sulfate glycosaminoglycans (HS-GAGs) represents a key phenomenon [54,55]. On the other hand, virus-susceptible cell interaction often requires several binding events to productive cell entry [56]. Cell surface HS-GAGs are co-receptors for several pathogens such as parasites, bacteria, and viruses [57]. For example different viruses, such as Sindbis virus [58], Venezuelan equine encephalitis virus [59], Adeno-associated virus type 2 [60], Foot-and-mouth disease virus [61], Herpesviruses [62,63,64,65], Human immunodeficiency virus [66], Echovirus [67], Dengue virus [51,68], Adenovirus types 2 and 5 [69], and Yellow fever virus [68], interact with GAGs. GAGs are linear polysaccharides that can be attached to proteins to form proteoglycans. There are five classes of GAGs: heparan-, chondroitin-, dermatan-, keratan sulfate, and hyaluronic acid. Heparan-sulfate, unlike other GAGs, is expressed in large quantities on most cell types and has been identified as an attachment factor for several viruses [70]. Concerning phlebovirus, it has been recently demonstrated that the first event in RVFV infection is the attachment to extracellular GAGs and that heparin, a GAG analog of HS, is able to inhibit virus-cell interaction [50].

Based on these observations, we have hypothesized that docking of TosV to cellular HS might be the initial step in the interaction between virus and the cell surface, and that bLf could exert its anti-TosV action by a competition for a common GAG receptor. To verify this hypothesis we first evaluated whether TosV infection could be neutralized either by heparin, a GAG analog heparan sulphate, or by removal of HS from the cell surface with different heparinases prior to infection. Our results demonstrated that HS-GAGs expressed on susceptible cell surface are involved in the binding of TosV, based on the following evidence: (i) binding and infection are competitively inhibited by pre-incubation of virus with heparin, a soluble receptor analog, in a dose-dependent manner, (ii) enzymatic cleavage of cell-surface HS-GAGs prevents infection.

Finally, to verify if bLf exerts its antiviral action by a competition for a common GAG receptor, experiments were carried out in which different concentrations of bLf and heparin, one at a time or mixed together, were incubated with Vero cells during the viral adsorption step. Our results showed that both bLf and heparin are able to prevent virus attachment to target cells when introduced one at a time. However, the antiviral effect of compounds was partially abolished when heparin and bLf were mixed together at specific concentrations, suggesting that they could interact with each other and that the anti-TosV effect of bLf is due to a direct competition for GAG receptors.

In conclusion, the results in this paper strengthen the knowledge that one way by which lactoferrin may inhibit infection of cells by certain viruses is through the blocking of HS-GAG virus receptors on the susceptible cell surface. Our findings, underlining the antiviral activity of bLf, may contribute to the development of an effective approach against TosV. TosV, endemic in the Mediterranean region, could disseminate to more temperate areas in Europe with abundant vectors, emphasizing the need to consider these viruses relevant from a European public health perspective. Moreover, new agents belonging to the Phlebovirus genus, the occurrences of which are probably due to genetic exchanges between different phleboviruses, and to climatic changes increasing vector abundance and distribution, have been recently discovered [71,72,73]. The antiviral action of bLf against TosV, highlighted in our study, could also be of help against emerging phleboviruses potentially implicated in severe human diseases.

## 4. Materials and Methods

### 4.1. Cells and Virus

Vero cells (ATCC CCL-81; an African greenmonkey kidney cell line) were grown at 37 °C in a humidified atmosphere, with 5% CO_2_ in Dulbecco’s modified Eagle’s medium (DMEM; Lonza, Milan, Italy) supplemented with 10% inactivated fetal calf serum (FCS, Flow Laboratories, Irvine, UK.), 2 mM glutamine, 2% non essential amino acids (Gibco, Paisley, UK), penicillin (100 IU/mL), and streptomycin (100 μg/mL).

TosV strain ISS.Phl.32 (isolated in 1981 from *Phlebotomus perniciosus* sand flies collected in Sesto Fiorentino, Florence, Italy) [74] was propagated in Vero cells and titrated by plaque assay. Briefly, after 1 h adsorption at 37 °C and 5% CO_2_, the inoculum was aspirated and the wells were overlaid with a mixture of one part 2% Gum Tragacanth and one part 2 × DMEM supplemented with 2.5% inactivated FCS and 2% 1M HEPES. The plates were incubated at 37 °C and 5% CO_2_ for 4–7 days, and then were stained with 1.5% crystal violet.

### 4.2. Chemicals

Lactoferrin from bovine milk (bLf), obtained from Morinaga Milk Industries (Zama City, Japan), was deprived of endotoxin as previously described [75]. Detoxified bLf was dissolved as a stock solution (0.25 mM) in pyrogen-free PBS. BLf purity was checked by SDS-PAGE stained with silver nitrate and was judged to be greater than 95%. Protein concentration was determined by UV spectroscopy on the basis of the extinction coefficient of 15.1 (280 nm, 1% solution) [76]. The iron saturation rate of bLf, determined by atomic absorption spectrometry, was approximately 19.4%.

Heparin (170 USP units/mg) was purchased from Sigma-Aldrich s.r.l. (Milan, Italy) and dissolved in pyrogen-free PBS to make a stock solution.

Heparinase I, II, and III from *Flavobacterium heparinum* were purchased from Sigma-Aldrich s.r.l. (Milan, Italy).

### 4.3. Cytotoxicity Assay

To establish the maximal non-cytotoxic dose of bLf, two-fold serial dilutions of protein in DMEM were incubated at 37 °C with confluent Vero cells grown in 96-well tissue culture microplates (Nalge Europe Ltd, Neerijse, Belgium). After 48 and 72 h, the following parameters were considered: cell morphology was examined by light microscopy, cell proliferation was evaluated quantitatively by microscopic counts after dispersion into individual cells with trypsin. Protein dilutions that did not affect any of these parameters were considered as non-cytotoxic concentrations and utilized for antiviral assays.

### 4.4. Action of bLf on TosV Cytopathic Effect

Vero cells grown in 96-well tissue culture microplates for 24 h at 37 °C in 5% CO_2_, were incubated with different concentrations of bLf before (1 h at 37 °C), and during virus adsorption (1 h at 37 °C). As viral inoculum was utilized TosV at a multiplicity of infection (m.o.i.) of 1 and 0.1 plaque forming unit (p.f.u.)/cell. Then, cells were rinsed thoroughly and incubated with the same concentrations of bLf at 37 °C in 5% CO_2_. The cytopathic effect (c.p.e.) induced by TosV was measured 48 h after infection by the neutral red uptake assay as previously described [38]. Briefly, treated and untreated infected cells were stained for 3 h with neutral red (50 μg/mL, 200 μL/well, 37 °C, 5% CO_2_); then the cells were washed with Hank’s salt solution and fixed for 10 min at room temperature (RT) with 4% formaldehyde, 10% CaCl_2_ (200 μL/well). The uptaken dye was extracted for 15 min at RT by 1% acetic acid in 50% ethanol (200 μL/well) and the disruption of the cells by the virus infection or the possible protection by the compounds were measured at 540 nm by an ELISA-reader. Results were expressed as percentage of c.p.e. inhibition by comparison with untreated infected control cultures.

### 4.5. Effect of bLf on Different Steps of TosV Infection

Vero cells, grown in 96-well tissue culture microplates for 24 h at 37 °C in 5% CO_2_, were infected with TosV (1 p.f.u./cell) for 1 h at 4 °C, then viral inoculum was removed, cell monolayers were washed three times with DMEM and incubated at 37 °C in 5% CO_2_. The c.p.e. induced by TosV was measured 48 h after infection. To ascertain whether the antiviral effect of bLf took place on viral adsorption or on a different step of viral replication, the inhibiting activity of 1 mg/mL bLf was assessed by following different experimental procedures: (i) the cells were incubated with bLf (1 h at 37 °C), washed three times with medium and than infected (1 h at 37 °C); (ii) bLf was added together with the virus inoculum during the adsorption step (1 h at 37 °C); (iii) bLf was incubated with the cells after the viral adsorption step (48 h at 37 °C); (iv) bLf was present during the whole experiment.

### 4.6. Inhibition of Infection by Heparin

Neutralization of virus binding to Vero cells was carried out by incubating serial fourfold heparin dilutions in culture medium with equal volumes (0.25 mL) of virus suspension containing 2.10^5^ p.f.u./0.25 mL. In negative controls, culture medium was used instead of heparin in the same volume. The mixtures were incubated for 1 h at 37 °C. Vero cells were seeded at 2 × 10^4^ in 96-well plates (Nalge Europe Ltd, Neerijse, Belgium) 24 h prior to infection. Medium was removed, and the cells were infected with 100 μL/well (in quadruplicate) of the virus-heparin mixtures. After 1 h adsorption at 37 °C, cells were rinsed thoroughly, and 200 μL of fresh medium was added to the cells, followed by incubation for 48 h at 37 °C. Virus infection was analyzed by measuring the c.p.e. by the neutral red uptake assay.

### 4.7. Enzymatic Removal of GAGs from the Surfaces of Vero Cells

Heparinase I, II, and III were reconstituted in 20 mM HEPES (pH 7.5), 50 mM NaCl, 4 mM CaCl_2_, and 0.01% bovine serum albumin (BSA). Dilutions were prepared in digestion buffer (20 mM HEPES pH 7.5, 150 mM NaCl, 4 mM CaCl_2_, 0.1% BSA) and various concentrations of the enzymes (100 μL/well) were added to Vero cells, which were then incubated for 1 h at 37 °C. Cells were then washed with the respective digestion buffers and were subsequently infected with TosV for 1 h at 37 °C. After the incubation, the cells were washed twice with serum-free medium, and infectivity was determined 48 h post-infection by neutral red uptake assay.

### 4.8. Immunofluorescence

Vero-infected cells were washed in PBS, fixed in acetone at −20 °C for 5 min, and incubated with affinity purified mouse hyperimmune ascitic fluids (MIAF) anti-Toscana virus, ISS.PHL3 prototype [4], for 45 min at 37 °C. After washing in PBS, viral antigen synthesis was estimated using FITC-conjugated anti-mouse gammaglobulin antibodies (Sigma-Aldrich s.r.l., Milan, Italy) and an UV Leitz microscope.

### 4.9. Statistical Analysis

Statistical analysis was performed by Student’s *t*-test for unpaired data. Data were expressed as the mean and SD and *p* values of <0.05 were considered significant.

## 5. Conclusions

Bovine lactoferrin strongly inhibits infection of susceptible cells by Toscana virus. The protein acts at the level of cell attachment, interfering with virus-cell binding through a competition for common glycosaminoglycan receptors. Our results provide additional insights into the antiviral efficacy of bovine lactoferrin suggesting a potential benefit of this protein against emerging phleboviruses.
